# Epidemiology, clinical history and microbiology of peritonsillar abscess

**DOI:** 10.1007/s10096-014-2260-2

**Published:** 2014-10-17

**Authors:** E. Mazur, E. Czerwińska, I. Korona-Głowniak, A. Grochowalska, M. Kozioł-Montewka

**Affiliations:** 1 Medical Microbiology Department, Medical University of Lublin, ul. Chodźki 1, 20-093 Lublin, Poland; 2 Department of Otolaryngology, Regional Specialist Hospital in Radom, ul. Aleksandrowicza 5, 26-617 Radom, Poland; 3 Department of Pharmaceutical Microbiology, Medical University of Lublin, ul. Chodźki 1, 20-093 Lublin, Poland; 4 Microbiological Laboratory, Regional Specialist Hospital in Radom, ul. Aleksandrowicza 5, 26-617 Radom, Poland

## Abstract

The purpose of this investigation was to explore the epidemiology, clinical history and microbiology of peritonsillar abscess (PTA). A retrospective review of PTA cases treated at the Department of Otolaryngology, Regional Specialist Hospital in Radom, Poland between 1st October 2003 and 30th September 2013 was undertaken. A total of 111 PTA patients were admitted. The study population consisted of 57.7 % males and 42.3 % females, with an average age of 31.0 (range 5–78) years. Smokers comprised 22.0 % of the study group. The seasonal variation of PTA was statistically insignificant (*p* = 0.45). Recurrent tonsillitis occurred in 35.5 % of patients. In comparison with the rest of the study population, patients with a history of recurrent pharyngotonsillitis had higher incidence of previous PTA episodes [odds ratio (OR) 17.8, 95 % confidence interval (CI) 2.1–148.7, *p* = 0.001]. Also, they were more frequently treated with antibiotics prior to hospitalisation (OR 4.6, 95 % CI 2.0–10.9, *p* = 0.0005) and had significantly longer hospital stay (*p* = 0.03). Bacterial cultures of abscess aspirates were performed in 40.5 % of patients. Monomicrobial growth was detected in 77.8 % of aerobic cultures. *Streptococcus pyogenes*, growing most frequently in monoculture, was found in 28.9 % of aerobic cultures. PTA patients with and without recurrent pharyngotonsillitis differed with regard to clinical history and course of disease. The percentage of smokers among PTA patients was lower than that described in the literature. Monomicrobial growth predominated in PTA aspirate cultures. *S. pyogenes* proved to be the most frequent pathogen.

## Introduction

Peritonsillar abscess (PTA), or quinsy, is defined as a collection of pus located between the tonsillar capsule and the pharyngeal constrictor muscle. Its aetiology and pathogenesis have not been fully elucidated [1]. It is considered to be a purulent complication of acute tonsillitis; however, other pathogenic mechanisms, such as obstruction of the supratonsillar Weber’s glands, have also been proposed [1–3]. Adolescents and young adults are the most commonly affected [4, 5]. Smoking proved to be associated with significantly increased risk of PTA [6]. There is no consensus as to whether or not seasonal variation in PTA incidence exists [2, 5, 7–9]. Moreover, there is much inconsistency regarding the microbiology of PTAs [1]. The majority of abscesses are polymicrobial infections, with three bacteria regarded to be key causative pathogens: *Streptococcus pyogenes*, *Fusobacterium necrophorum* and *Streptococcus milleri* group [1, 4, 7, 10–13]. Monomicrobial infections, however, have been reported as well [14, 15]. Management protocols are lacking, and treatment methods include needle aspiration, surgical drainage or quinsy tonsillectomy combined with antimicrobial therapy [5, 16, 17]. Since data regarding PTA in Poland are scarce [18], the aim of our work was to explore the PTA epidemiology, clinical history and microbiology. 

## Patients and methods

A retrospective study was performed on PTA cases treated at the Department of Otolaryngology, Regional Specialist Hospital in Radom, Poland between 1st October 2003 and 30th September 2013. Radom has a population of 220,000 people and is located in central Poland. The hospital is the only one in the city providing full-term otolaryngological medical care. To perform aerobic cultures, abscess aspirates were inoculated on sheep blood, chocolate, mannitol salt, MacConkey and Sabouraud agar plates, and incubated in a carbon dioxide-enriched atmosphere. Anaerobic cultures were carried out with the usage of Schaedler agar plates. The media were incubated for up to 3 days at 37 °C, with the exception of Sabouraud agar plates, which were incubated at 30 °C. The identification of species was carried out with the use of routine microbiological methods. The medical records of all PTA patients were reviewed to obtain the following data: age, sex, previous episodes of abscess, past history of recurrent tonsillitis, seasonality, laterality, smoking and dental status, antibiotic treatment proceeding admission to the hospital, length of hospitalisation, neutrophil count and C-reactive protein value, and results of microbiological examination of PTA purulent contents. A past history of recurrent tonsillitis has been specified as three or more, five or more, or seven or more tonsillitis episodes within the previous 1, 2 or 3 years, respectively. The study was approved by the Bioethics Committee at the Medical University of Lublin, Poland.

### Statistical analysis

Data processing and analysis were performed using STATISTICA version 10 (StatSoft, Inc.). The results were expressed as percentage or mean, median (range). The association between each independent variable was statistically analysed in different groups. Continuous variables were compared using the non-parametric test (Mann–Whitney *U*-test) and categorical variables by Pearson’s Chi-squared or Fisher’s exact test, as appropriate. Odds ratios (ORs) and their 95 % confidence intervals (CIs) were calculated. Statistical significance was set at *p* < 0.05.

## Results

**Fig. 1:**
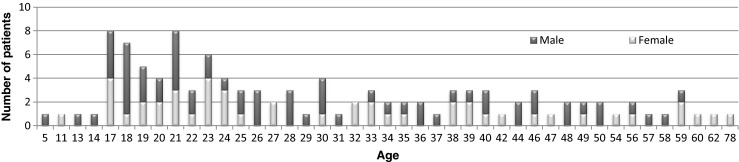
Age and sex distribution among patients with peritonsillar abscess (PTA) hospitalised at the Department of Otolaryngology, Regional Specialist Hospital in Radom, Poland between 1st October 2003 and 30th September 2013

A total of 111 patients were admitted over the 10-year study period. The study population consisted of 57.7 % males and 42.3 % females, with an average age of 29.7 ± 12.9 years (range 5–59 years) and 32.7 ± 15.2 years (range 11–78 years), respectively. The main procedure undertaken was diagnostic needle aspiration, followed by abscess incision and drainage combined with antibiotic therapy. Cefuroxime and metronidazole were administered as empiric antimicrobial therapy in most cases. The age and sex distribution among PTA patients is illustrated in Fig. 1. Table 1 depicts the clinical and epidemiological characteristics of the study population.

**Table 1:** Clinical and epidemiological characteristics of the study population

Characteristic		*n* = 111
Mean, median (range) age		31.0, 27.0 (5–78 years)
Gender	Female	47 (42.3)
	Male	64 (57.7)
Dental status^a^	Healthy teeth (1)	24 (24.0)
	Filled teeth (2)	41 (41.0)
	Single cavities (3)	24 (24.0)
	Dental caries (4)	11 (11.0)
Smokers^a^		22 (22.0)
Recurrent pharyngotonsillitis in the past^b^		39 (35.5)
Abscess side/laterality	Left	68 (61.3)
	Right	41 (36.9)
	Bilateral	2 (1.8)
Previous PTA episodes		9 (8.1)
Seasonal presentation	Winter	25 (22.5)
	Spring	26 (23.4)
	Summer	33 (29.7)
	Autumn	27 (24.3)
Mean, median (range) hospital stay		5.0, 5.0 (1–17 days)
Mean, median (range) CRP value (mg/L)^c^		101.8, 77.3 (2.0−338.0)
Mean, median (range) WBC value (×10^3^/μl)^d^		15.2, 14.7 (4.4−61.3)
Antibiotic therapy prior to admission		52 (46.8)
Comorbidities		30 (27.0)

Values are numbers (%), unless stated otherwise

*PTA* peritonsillar abscess, *CRP* C-reactive protein, *WBC* white blood cells

^a, b, c, d^Data available for 100, 110, 93 and 105 patients, respectively

The seasonal variation for PTA incidence was statistically insignificant (*p* = 0.45, Pearson’s Chi-squared test). There was no significant association between smoking habits and age among PTA patients (*p* = 1.0, OR 0.9, 95 % CI 0.3–2.8, Fisher’s exact test). In the group of patients younger than 40 years old, the percentage of smokers was comparable to that in the older age group (22.4 % versus 20.8 %). Men were more frequently smokers than women (27.1 % versus 14.6 %), but without reaching statistical significance (*p* = 0.15, OR 2.2, 95 % CI 0.8–6.1, Fisher’s exact test).

Thirty (27.0 %) patients had comorbidities; cardiovascular (36.7 %), neurologic (13.3 %) and thyroid (13.3 %) disorders proved to be the most frequent ones. Other comorbidities included diabetes mellitus, liver and ophthalmic disorders, peptic ulcer disease, rheumatoid arthritis, leukaemia and Wilson’s disease. When the PTA patients were divided into two age groups (<40 years old and older), it turned out that, in older patients, who comprised 24.3 % (27/111) of the study group, comorbidities occurred more frequently (OR 4.5, 95 % CI 1.8–11.4, *p* = 0,0022, Fisher’s exact test) and the dental status was worse (OR 2.8, 95 % CI 1.1–7.4, *p* = 0.044, Fisher’s exact test).

The clinical and epidemiological characteristics of patients with a history of recurrent pharyngotonsillitis were compared with the rest of the study population (Table 2).

**Table 2:** Clinical and epidemiological characteristics of patients with a history of recurrent pharyngotonsillitis compared with the rest of the study population

Characteristic		Recurrent pharyngotonsillitis in the past (% in group)		*p*-Value
		Yes (*n* = 39)	No (*n* = 71)	
Age <40 years		32 (82.1)	51 (71.8)	0.25
Male		21 (53.8)	42 (59.2)	0.69
Smokers		6 (16.7)	16 (25.4)	0.45
Dental status 3–4		15 (40.5)	20 (32.3)	0.52
Left side PTA		26 (66.7)	41 (59.4)	0.54
Previous PTA episodes		8 (20.5)	1 (1.4)	0.001*
Seasonal presentation:	Winter	11 (28.2)	14 (19.7)	0.61
	Spring	10 (25.6)	16 (22.5)	
	Summer	11 (28.2)	22 (31.0)	
	Autumn	7 (18.0)	19 (26.8)	
Median (range) hospital stay		5.0 (1−12)	4.0 (1−17)	0.03*
Median (range) CRP value (mg/L)		95.6 (3.5−300.0)	73.0 (6.1−338.0)	0.52
Median (range) WBC value (×10^3^/μl)		14.6 (9.0−61.3)	14.7 (4.4−35.2)	0.77
Antibiotic therapy prior to admission		27 (69.2)	25 (35.2)	0.0005*
Comorbidities		12 (30.8)	18 (25.4)	0.66

*PTA* peritonsillar abscess, *CRP* C-reactive protein, *WBC* white blood cells

*Statistically significant

In 45 patients (40.5 %), bacterial cultures of abscess aspirates were performed. In 19 of them, both aerobic and anaerobic cultures were carried out. In this group, a mixture of aerobic and anaerobic bacteria was detected in two specimens, and only anaerobic bacteria (*Prevotella oralis*) or only aerobic bacteria were detected in one and 16 samples, respectively. Monomicrobial growth was observed in samples obtained from 14 out of 19 patients (73.7 %), and two or three isolates were yielded in samples collected from three and two patients, respectively. In the remaining 26 patients, only aerobic culture was performed. Monomicrobial growth was observed in samples obtained from 21 out of 26 patients (80.8 %). In samples collected from five patients, two isolates were yielded.

In nine out of 45 aerobic cultures (20.0 %), polymicrobial growth was observed. In 15 aerobic cultures (33.3 %), only species belonging to normal oropharynx flora were detected, namely *Streptococcus mitis* (one isolate), *Streptococcus oralis* (four isolates), *Streptococcus salivarius* (three isolates), *Streptococcus sanguinis* (one isolate) and other viridans streptococci, excluding those belonging to *Streptococcus milleri* group (ten isolates). Potential pathogens were identified in 29 aerobic cultures (64.4 %). The most frequently isolated pathogen proved to be *S. pyogenes*, found in 13 out of 45 aerobic cultures (28.9 %). It was most frequently isolated as monoculture (10/13, 76.9 %). In three cultures, *S. pyogenes* was detected as one of two isolates, together with *Enterococcus* sp., *Escherichia coli* and *S. salivarius*, respectively. Microbial species isolated from PTA aspirate aerobic and anaerobic cultures are shown in Table 3.

**Table 3:** Microbial species isolated from peritonsillar abscess (PTA) aspirate aerobic and anaerobic cultures

Species	No. of positive cultures
Aerobic	44/45
*Streptococcus pyogenes*	13
*Streptococcus* C group	1
*Streptococcus agalactiae*	1
*Streptococcus anginosus*	4
*Streptococcus constellatus*	3
*Streptococcus* viridans group^a^	15
*Enterococcus* sp	2
*Staphylococcus aureus*	1
*Haemophilus influenzae*	1
*Escherichia coli*	1
*Acinetobacter* sp.	2
*Candida albicans*	2
Anaerobic	3/19
*Fusobacterium necrophorum*	1
*Prevotella oralis*	2
*Prevotella denticola*	1

^a^Excluding *Streptococcus milleri* group

Among 45 patients in whom bacterial cultures of abscess aspirates were performed, 25 (55.6 %) received antibiotic therapy prior to admission. Differences were observed in the isolation of potential pathogens between patients with and without prior antibiotic therapy, although they were not statistically significant. However, from patients who did not receive antibiotic therapy prior to admission, potential pathogens were isolated more frequently than normal oropharynx flora (OR 4.5, 95 % CI 1.0–19.2, *p* = 0.05, Fisher’s exact test). Also, when *S. pyogenes* was analysed separately from other potential pathogens, it turned out that a significantly higher incidence of its isolates were observed in patients without prior antibiotic therapy (OR 2.1, 95 % CI 1.1–4.0, *p* = 0.037, Fisher’s exact test).

Two patients (1.8 %) have bilateral PTA. Both were treated with antibiotics before admission to the hospital. They did not have any comorbidities; however, one of them was in early pregnancy at the time of hospitalisation. In only one of them was aerobic culture of abscess purulent contents performed and normal oropharynx flora isolated.

All but two patients recovered without complications. In one of them, with left-side PTA and *S. pyogenes* detected in abscess pus, neck phlegmon occurred. In the second patient, with left-side PTA and both *Acinetobacter baumannii* and *Enterococcus faecalis* isolated from abscess aspirate aerobic culture, neck phlegmon and sepsis developed. Both patients were transferred to the Intensive Care Department and, after being treated there, recovered.

## Discussion

This study characterises several facets of PTA epidemiology, as well as its clinical history and microbiology.

PTA is a disease usually affecting teenagers and young adults; its incidence is rare in infants and children. The majority of studies report male predominance; however, equal male-to-female ratios have been described as well [5, 7, 8, 10, 12, 13, 19–23]. In our series, the male-to-female ratio proved to be 1.4:1 and the majority of cases occurred in patients at the age between 17 and 30 years. Patients aged 40 years or older comprised 24.3 % of the study group. Similar percentages of older patients, namely 24.4 % and 25 %, were reported by Marom et al. [7] and Matsuda et al. [21], respectively. We noticed that, in this age group, comorbidities occurred significantly more frequently and the dental status of patients proved to be significantly worse as compared with patients younger than 40 years old. Among our patients, the left tonsil proved to be affected more frequently. A similar laterality distribution was found in the studies carried out in Spain and Northern Ireland [19, 22]. However, equal numbers of left and right PTA have also been reported [5, 7]. The average incidence of bilateral PTA has been estimated to be 4.8–4.9 % [24]. In our series, bilateral PTA was found in 1.8 % of patients. A similar rate of bilateral PTA, namely 1.0 %, was detected in the study conducted in Singapore [5], whereas other works reported bilateral PTA in up to 0.8 % of patients [7, 9, 19]. Previous PTA episodes were present in 8.1 % of our study population, whereas other studies reported them in between 11 % and 16 % of their patients [7, 19, 22]. In our series, previous PTA episodes proved to occur more frequently in patients with a history of recurrent pharyngotonsillitis (OR 17.8, 95 % CI 2.1–148.7, *p* = 0.0011).

Although seasonal variation in PTA incidence has been reported previously, there was no consensus among studies with respect to the seasonal trend direction. Winter, spring and autumn peaks were observed [2, 5, 7, 9]. By contrast, in a recent study carried out in Denmark [8], as well as in our work, the seasonal variation of PTA proved to be statistically insignificant.

Smoking habits among PTA patients were analysed in several studies [6, 7, 13, 25, 26]. In a recent Danish study, smoking proved to be associated with significantly increased risk of PTA in both males and females [6]. Marom et al. [7], Klug et al. [6] and Hidaka et al. [13] reported, respectively, 33.7 %, 36 % and 69 % of smokers among PTA patients. According to Dilkes et al. [25], PTA patients are 70 % more likely to smoke than the general population. Surprisingly, in our series, smokers comprised only 22.0 % of the study group. However, this result may have been biased by the fact that data regarding smoking status were available for 90.1 % of our patients. Moreover, only current smoking status declared by patients was involved in the patient records. For most of them, the information about past smoking habits was not provided. Klug et al. [6] found that smoking PTA patients are older than non-smoking patients; however, we did not find a significant association between smoking habits and age among our study population (*p* = 1.0, OR 0.9, 95 % CI 0.3–2.8).

Recurrent tonsillitis has been considered to be important in PTA pathogenesis [5]. However, it was reported to be present in the medical history of 12–37.4 % of PTA patients [5, 9, 12, 19]. In our study, the percentage of patients with recurrent tonsillitis proved to be 35.5 %. In this group, we noted a significantly higher incidence of previous PTA episodes as well as longer hospital stay in comparison with the remaining patients. Also, among patients with recurrent tonsillitis, significantly more cases were treated with antibiotics prior to hospitalisation in comparison with the rest of the study population. Recently, Powell et al. [1] hypothesised that there are presumably two pathogenically distinct PTA subtypes. Type 1 occurs in patients without recurrent pharyngotonsillitis and contains a pure culture of a single organism, most frequently *S. pyogenes*. Type 2, in turn, is associated with a history of recurrent tonsillitis and displays polymicrobial growth, often containing anaerobes. Type 2 has more severe clinical presentation, associated with chronic underlying microfloral imbalance due to previous antibiotic use. Our findings show that PTA patients with and without recurrent tonsillitis differed with regard to clinical history and course of disease. We did not find differences in the microbiological profile of aspirate cultures among patients with and without recurrent tonsillitis; however, this could be due to the fact that microbiological examination was performed in only 40.5 % of the study population.

The majority of reported PTAs are polymicrobial infections [1, 4, 7, 10–13]. However, in the work carried out by Snow et al. [15], 72.3 % of patients proved to have monomicrobial infection. Also, Megalamani et al. [14] identified monomicrobial growth in 65 % of their aerobic cultures. In our work, monomicrobial growth in PTA cultures predominated as well. It was observed in samples obtained from 73.7 % of patients in whom both aerobic and anaerobic cultures were performed, as well as from 80.8 % of those in whom only aerobic culture was carried out. However, since complete microbiological examination of PTA aspirates was performed in only a small number of our patients, these results should be interpreted with caution.

Three bacteria are regarded to be key causative pathogens in PTA: *Streptococcus pyogenes*, *Fusobacterium necrophorum* and *Streptococcus milleri* group, including *S. intermedius*, *S. anginosus* and *S. constellatus* [1, 4, 7, 10–13]. We considered *Streptococcus viridans* other than *S. milleri* group to be normal oropharynx flora and all other bacterial species to be potential pathogens. *S. pyogenes*, one of the most prominent PTA pathogens, proved to be isolated from between 20 % and 30 % or approximately 45 % of PTAs [1, 7, 10, 12, 20]. It was growing more frequently as a sole isolate than in mixed culture [14, 15, 20, 22, 27]. In our work, *S. pyogenes* growing most often as monoculture was found in 28.9 % of aerobic cultures. It proved to be the most frequently isolated pathogen, followed by *S. anginosus*, *S. constellatus* and *Prevotella* sp. However, viridans streptococci other than *S. milleri* group were isolated from 35.5 % of aerobic cultures. Since PTA patients are frequently at different stages of antimicrobial therapy at the time of presentation (46.8 % of our patients were), abscess aspirate cultures may not necessarily represent the actual causative organism(s). Some studies reported no significant difference in the cultured bacteria between patients with and without prior antibiotic treatment [14, 23, 28]. In our work, differences observed in the isolation of potential pathogens between patients with and without prior antibiotic therapy were not statistically significant as well. However, potential pathogens were isolated more frequently than normal oropharynx flora from patients who did not receive antibiotic therapy prior to admission (OR 4.5, 95 % CI 1.0–19.2, *p* = 0.05). Also, we observed a significantly higher incidence of *S. pyogenes* isolates in patients without antibiotic therapy prior to PTA presentation, which is in accordance with several previous reports [7, 20, 29, 30]. Thus, it is possible that *S. pyogenes* can be the causative pathogen in many more PTA cases than those in which it has been isolated in abscess aspirate culture. On the other hand, however, the percentages of *S. pyogenes* isolations in patients without prior antibiotic treatment in the study carried out by Megalamani et al. [14] proved to be similar to those observed in patients who were treated with antibiotics before PTA presentation.

Summarising, we found that PTA patients with and without recurrent pharyngotonsillitis differed with regard to clinical history and course of disease. The percentage of smokers was lower than that described in the literature. Monomicrobial growth predominated in PTA aspirate cultures. *S. pyogenes* proved to be the most frequent pathogen. The strength of our study is the careful analysis of epidemiological, clinical and microbiological data in a relatively large group of PTA patients. However, it has several limitations. One of them is its retrospective nature. Moreover, bacteriologic examination was carried out in a small number of patients, and in an even smaller percentage it was complete, i.e. both aerobic and anaerobic cultures of PTA aspirate were performed. Thus, solid conclusions regarding PTA microbiology are difficult to draw. Prospective studies, carried out according to a unified protocol including complete bacteriologic PTA aspirate examination, would be indicated in order to find out whether or not there are differences regarding the microbiological profile in patients with and without a history of recurrent pharyngotonsillitis.
